# Effectiveness of Delivery Room Continuous Positive Airway Pressure in Term Neonates With Respiratory Distress: A Single-Center Experience

**DOI:** 10.7759/cureus.99110

**Published:** 2025-12-13

**Authors:** Prajwal B Gadgeesh, Vikram Sakaleshpur Kumar, Niveditha Hegde Venkatramana, Latha S P

**Affiliations:** 1 Pediatrics and Neonatology, Subbaiah Institute of Medical Sciences, Shivamogga, IND; 2 Pediatrics and Child Health, Subbaiah Institute of Medical Sciences, Shivamogga, IND; 3 Pediatric Medicine, Sarji Mother and Child Care Hospital, Shivamogga, IND; 4 Pediatrics, Subbaiah Institute of Medical Sciences, Shivamogga, IND

**Keywords:** continuous positive airway pressure, delivery room cpap, infant, neonatal intensive, newborn, noninvasive ventilation, respiratory distress syndrome, term neonate

## Abstract

Background

Neonatal intensive care unit (NICU) admission can represent a critical period for full-term newborns and may reflect underlying health needs. Continuous positive airway pressure (CPAP), a noninvasive method of respiratory support, is now widely used to prevent and treat respiratory distress. CPAP aims to minimize reliance on mechanical ventilation, allow earlier intervention, and reduce both NICU admissions and overall hospital stay.

Methods

This retrospective descriptive study was conducted over seven months (November 2023 to May 2024) at a tertiary care hospital. Inborn, spontaneously breathing term neonates (gestational age ≥36 weeks + 6 days to <41 weeks + 6 days) with a Modified Downe’s score >3 and <7 at birth were included. Neonates with major anomalies, intubation at birth, prolonged positive-pressure ventilation, or genetic syndromes were excluded. Delivery room CPAP (DR-CPAP) was applied at FiO₂ 30% (titrated) and PEEP 5 cm H₂O for up to 30 minutes. The primary outcome was improvement or deterioration of labored breathing, as measured by the Modified Downe’s score, after DR-CPAP. Secondary outcomes included duration of respiratory support, NICU stay, and complications (e.g., air-leak syndrome). Data were analyzed using IBM SPSS Statistics for Windows, Version 26.0 (Released 2018; IBM Corp., Armonk, NY, USA): continuous variables were reported as mean ± SD, categorical variables as frequencies (%), and associations tested using chi-square or t-tests, with p < 0.05 considered significant. Given a 20% expected NICU admission rate, a sample size of 90 neonates provided approximately 80% power to detect a 15% difference at α = 0.05.

Results

Ninety-three neonates were included (male: 49 (53%); female: 44 (47%)). Seventy-one of 93 (76%) recovered within 30 minutes of DR-CPAP, while 22 (24%) required NICU admission; none required mechanical ventilation. Among admitted neonates, only seven (7.5% of the total) required CPAP, and the remaining 15 received free-flow oxygen via hood or nasal prongs. No complications, such as pneumothorax, were observed. A significant association was found between delayed initiation of breastfeeding (>1 hour) and NICU admission (p < 0.01). Gestational age, sex, meconium-stained liquor, weight at admission, and mode of delivery were not significantly associated.

Conclusions

Our study demonstrates that DR-CPAP is an effective intervention for term neonates experiencing respiratory distress syndrome. This approach significantly reduced the need for subsequent mechanical ventilation and the duration of NICU stay in our single-center experience. The early application of CPAP immediately post-delivery can be safely implemented in resource-limited settings to improve neonatal outcomes.

## Introduction

Admission to the neonatal intensive care unit (NICU) for full-term newborns is a significant event that raises concerns about potential mortality risk extending into childhood [1]. Efforts to reduce NICU admissions for full-term infants born at or after 37 weeks are essential to prevent avoidable harm. Over 20% of full-term NICU admissions could be avoided, highlighting the importance of keeping mother and baby together to minimize the negative effects of separation after birth [2].

Advances in neonatal intensive care have progressed alongside improvements in the treatment of respiratory failure in preterm infants. Ventilatory support now ranges from gentler continuous positive airway pressure (CPAP) to various mechanical ventilation modes, including high-frequency ventilation. The introduction of less invasive CPAP delivery methods has enabled prompt intervention for infants with respiratory distress syndrome (RDS), reducing the need for mechanical ventilation, allowing earlier treatment initiation, and decreasing both NICU admissions and hospital stays.

Building on this progress, delivery room CPAP (DR-CPAP) has emerged as a promising intervention capable of preventing invasive ventilation and reducing long-term morbidity. Early initiation of noninvasive ventilation in the delivery room is a key strategy to optimize respiratory support for preterm infants. Studies highlight the benefits of noninvasive ventilation over invasive methods, emphasizing its role in reducing the need for intubation and mechanical ventilation, which are associated with increased mortality and morbidity [3]. However, while CPAP is well established in preterm populations, evidence for its use in term neonates remains limited and controversial, particularly due to concerns regarding the risk of pneumothorax [4,5].

The Neonatal Resuscitation Program (NRP) now recommends considering CPAP for spontaneously breathing infants with labored breathing or hypoxia in the delivery room, regardless of gestational age. CPAP supports the establishment of functional residual capacity (FRC) and improves ventilation-perfusion matching during the transition to extrauterine life. Although recent studies have raised concerns about DR-CPAP in late preterm and term infants, noting a higher risk of pneumothorax due to physiological differences [4,5], based on prior institutional experience, our center adopted a policy to use CPAP for all neonates with respiratory distress at birth, irrespective of gestational age. Here, we share our experience using CPAP in term neonates with respiratory distress.

The overall aim of this study was to evaluate the effectiveness and safety of DR-CPAP in term neonates presenting with moderate respiratory distress in preventing subsequent NICU admissions. The specific objectives were (1) to assess the primary outcome, improvement or deterioration in labored breathing using the Modified Downes score following DR-CPAP intervention, and (2) to evaluate secondary outcomes, including the duration of respiratory support, length of NICU stay for admitted neonates, and the occurrence of complications, specifically air-leak syndromes, within the study population.

## Materials and methods

Study design and setting

This was a single-center, retrospective descriptive study conducted at a tertiary care hospital. Data were collected from hospital records over a seven-month period, from November 2023 to May 2024.

Study population and selection criteria

Inborn, spontaneously breathing term neonates (gestational age >36 weeks + 6 days to <41 weeks + 6 days) presenting with labored breathing and moderate respiratory distress were included. The severity of respiratory distress was assessed using a modified Downes score, which evaluates five clinical parameters, such as cyanosis, respiratory rate, retractions, grunting, and air entry, each graded 0-2, for a maximum possible score of 10. In this study, moderate respiratory distress was defined as a modified Downes score greater than 3 and less than 7 [6].

Neonates were excluded if they had antenatal or postnatal diagnoses of congenital respiratory anomalies or critical congenital heart diseases, required major surgical interventions, were intubated at birth, required prolonged positive-pressure ventilation (defined as >1 minute), or had known genetic syndromes.

Clinical workflow and data collection

Neonates received DR-CPAP with 30% FiO₂ (titrated to achieve target oxygen saturation) and positive end-expiratory pressure (PEEP) of 5 cm H₂O using a T-piece resuscitator (Neopuff Infant Resuscitator, Fisher & Paykel Healthcare, Auckland, New Zealand). The initial modified Downes score was assessed immediately upon the neonate’s arrival in the resuscitation bay (within the first minute of life) and reassessed every five minutes during the 30-minute intervention period.

Data were retrospectively extracted from hospital records and physical charts using a standardized data collection form. Variables included gestational age, sex, initial Downes score, duration of CPAP, duration of NICU stay, and any recorded complications.

Assessment and intervention were performed by advanced NRP-trained junior resident doctors, with confirmation by senior residents. DR-CPAP was administered for a maximum of 30 minutes. If labored breathing worsened during this period, DR-CPAP was discontinued, and an alternative management plan was initiated according to institutional guidelines. Therapy was weaned and switched to routine care if the neonate’s respiratory distress resolved within 30 minutes. Stable, spontaneously breathing term neonates who did not meet NICU admission criteria were transferred to the postnatal ward, where they roomed in with their mothers and received routine newborn care and daily pediatric assessments. Our facility follows a rooming-in model rather than a separate transitional or well-baby nursery.

Outcome measures

The primary outcome was improvement or deterioration in labored breathing, assessed using the Modified Downes score following DR-CPAP intervention. Secondary outcomes included duration of respiratory support, NICU stay, and occurrence of complications, specifically air-leak syndromes. For neonates admitted to the NICU for further respiratory management, chest X-rays were performed to rule out air-leak syndrome, which was operationally defined as a confirmed diagnosis of pneumothorax or pulmonary interstitial emphysema on chest X-ray.

Statistical analysis

Data were analyzed using IBM SPSS Statistics for Windows, Version 26.0 (Released 2018; IBM Corp., Armonk, NY, USA). Continuous variables are presented as mean ± SD, and categorical variables as counts and percentages. Associations between categorical variables were tested using chi-square tests and between continuous variables using t-tests. A p-value < 0.05 was considered statistically significant. Effect size for significant associations was quantified using Cramér’s V. CIs (95% CI) for proportions were calculated using the Wilson method. Assuming a 20% expected NICU admission rate, a sample size of approximately 90 provided ~80% power to detect a 15% difference at α = 0.05.

## Results

**Figure 1:**
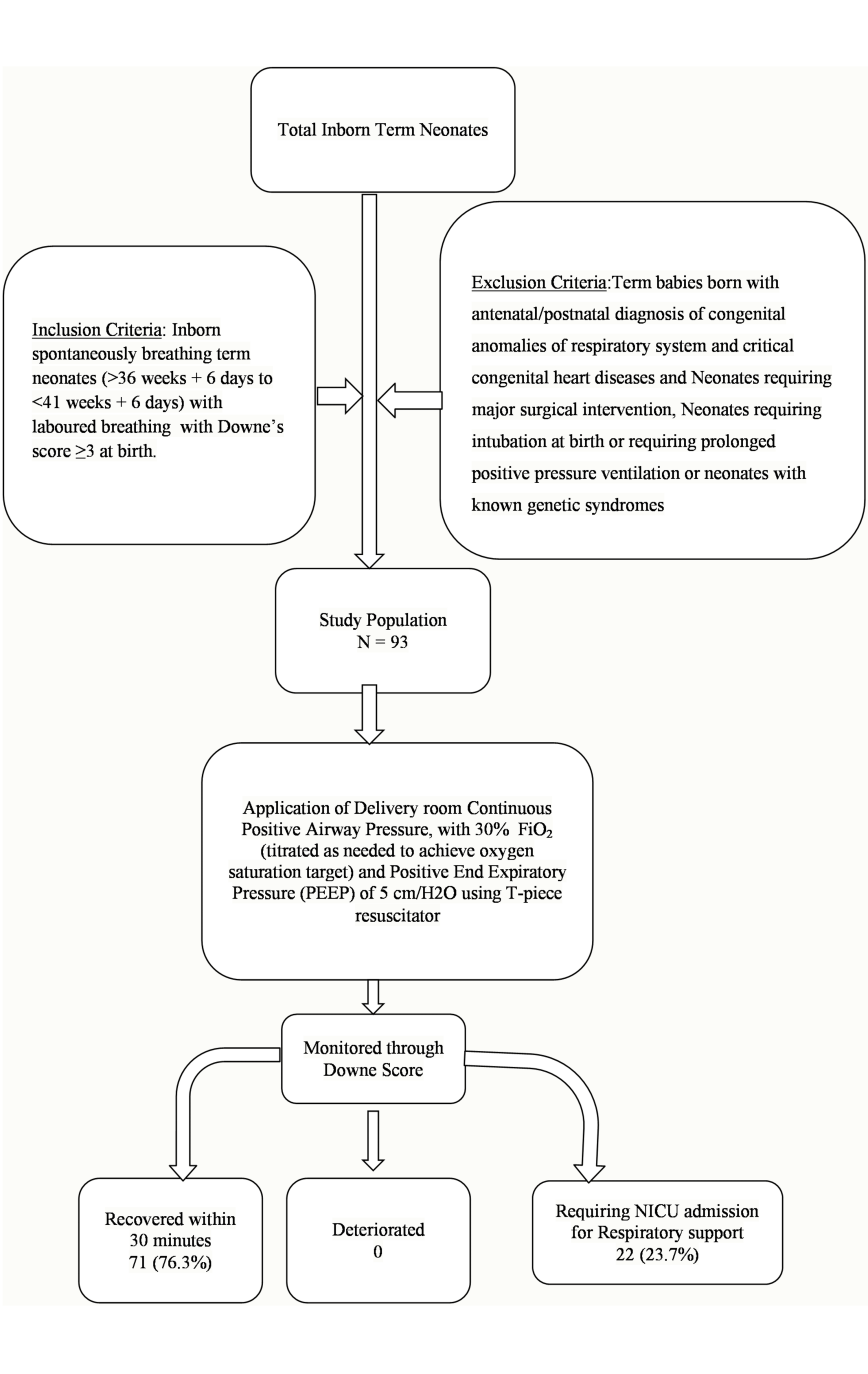
Flowchart illustrating the research methodology and primary outcomes assessed in the study CPAP, continuous positive airway pressure; NICU, neonatal intensive care unit; PEEP, positive end expiratory pressure

**Figure 2:**
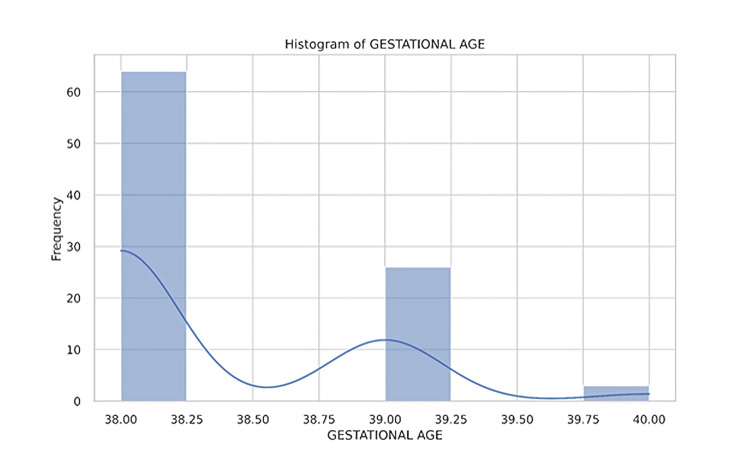
Distribution of neonates by gestational age

**Figure 3:**
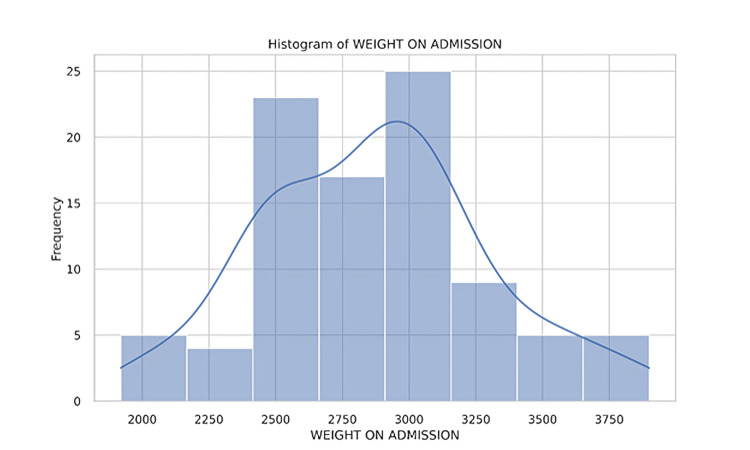
Distribution of neonates by birth weight

Ninety-three neonates were identified after applying the inclusion and exclusion criteria for inborn neonates over a seven-month period using the hospital neonatal data registry (Figure 1). Of these, 44 (47%) were female, and 49 (53%) were male. The majority of deliveries were via cesarean section (LSCS), with a smaller number delivered through full-term normal vaginal delivery or assisted normal vaginal delivery. All neonates were born at term, with a gestational age of 38-40 weeks (Figure 2). Birth weights ranged from 1,920 grams to 3,900 grams (Figure 3), with several neonates classified as small for gestational age (SGA) or large for gestational age (LGA) (Table 1).

**Table 1:** Demographic and clinical characteristics of the study population (n = 93) FTNVD, full-term normal vaginal delivery; LSCS, lower segment cesarean section; NVD, normal vaginal delivery

Variable	Category	n (%)
Sex	Male	49 (52.7%)
	Female	44 (47.3%)
Gestational age (weeks)	38	64 (68.8%)
	39	26 (28.0%)
	40	3 (3.2%)
Birth weight (g)	2000-2500	21 (22.6%)
	2500-3000	38 (40.9%)
	3000-3500	26 (28.0%)
	3500-4000	9 (9.7%)
Mode of delivery	LSCS	69 (74.2%)
	FTNVD	21 (22.6%)
	Assisted NVD	3 (3.2%)
Meconium-stained liquor	Present	13 (14.0%)
	Absent	80 (86.0%)
Breastfeeding within one hour	Yes	80 (86.0%)
	No	13 (14.0%)

A significant number of neonates required respiratory support in the form of CPAP. The majority had good Apgar scores, with most scoring 9/10 or 10/10 at 1 and five minutes. Most neonates were breastfed within the first hour of life, indicating early initiation of breastfeeding. Vital signs, including heart rate, respiratory rate, SpO₂, and temperature, were generally within the normal range for newborns.

Hospital length of stay ranged from 0 to 28 days, with most neonates not requiring NICU admission. The most common diagnosis was transient tachypnea of the newborn, with some neonates also presenting with conditions such as meconium aspiration syndrome, SGA, LGA, neonatal sepsis, and intrauterine growth restriction.

**Figure 4:**
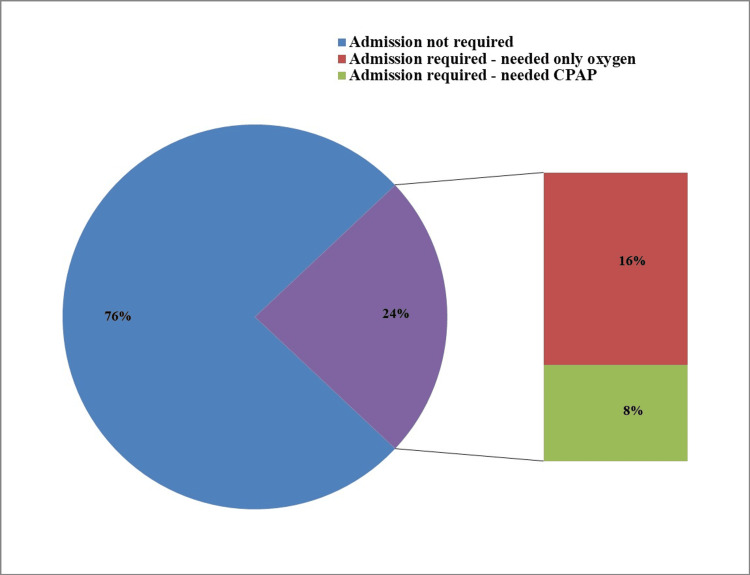
Frequency of NICU admission and respiratory support modalities This figure shows the distribution of neonates based on the need for NICU admission. Among the 24% (n = 22) who required admission, 8% (n = 7) received CPAP, while 16% (n = 15) received oxygen only. CPAP, continuous positive airway pressure; NICU, neonatal intensive care unit

Of the 93 neonates, 71 (76%) recovered within 30 minutes of DR-CPAP, while 22 (24%) required NICU admission for respiratory support (Figure 4). Among the admitted neonates, seven required CPAP support, representing a small proportion (8% of the total population), while the remaining 15 (16%) received free-flow oxygen via hood or nasal prongs. No complications were observed with DR-CPAP in this study.

Overall, 71 of 93 neonates (76.3%; 95% CI: 67.0-84.0%) recovered within 30 minutes of DR-CPAP, whereas 22 (23.7%; 95% CI: 16.0-33.0%) required NICU admission. Among neonates with delayed breastfeeding initiation (>1 hour), NICU admission occurred in 12 of 13 (92.3%; 95% CI: 64.0-99.8%), compared with 10 of 80 (12.5%; 95% CI: 6.2-21.6%) in the early breastfeeding group (p < 0.01).

**Figure 5:**
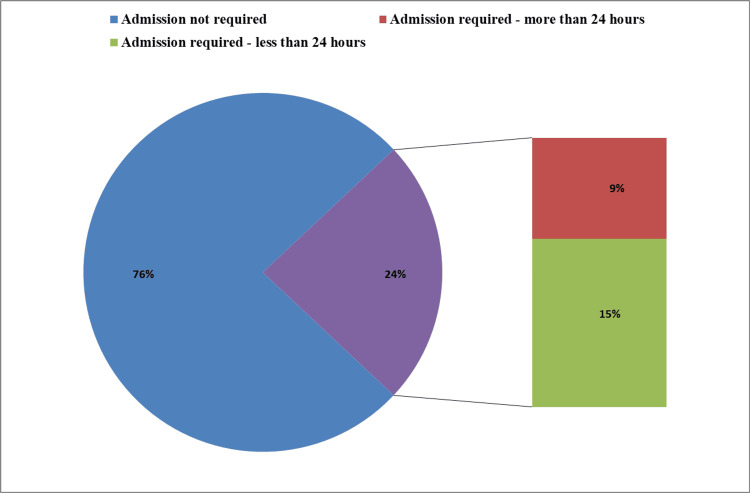
Distribution of neonates based on the duration of NICU admission requirements This figure shows the percentage distribution of neonates based on NICU admission and the duration of stay. Data are presented as percentages (%). Seventy-six percent of neonates did not require NICU admission. Of the 24% (n = 21) who required admission, 9% (n = 8) stayed for more than 24 hours, and 15% (n = 13) stayed for less than 24 hours. NICU, neonatal intensive care unit

The study found that factors such as gestational age, sex, meconium-stained liquor, admission weight, and mode of delivery were not significantly associated with the need for respiratory support. However, delayed initiation of breastfeeding was significantly associated with NICU admission; neonates requiring respiratory support experienced markedly delayed breastfeeding initiation. These findings highlight the importance of minimizing NICU admissions to support early breastfeeding. Most neonates who required NICU admission had a stay of less than 24 hours (Table 2, Figure 5).

**Table 2:** Characteristics of study population stratified by NICU admission Data are presented as numbers (N) and percentages (%). P-values were calculated using the chi-squared (χ²) test. Statistical significance was defined as p < 0.05. The relevant χ² test statistic and corresponding p-value are included for each parameter. FTNVD, full-term normal vaginal delivery; LSCS, lower segment cesarean section; NICU, neonatal intensive care unit; NVD, normal vaginal delivery

Parameter	No NICU admission (n = 71)	NICU admission (n = 22)	Total	χ²	p-Value
Gestational age (weeks)				1.08	0.58
38	49 (69%)	15 (68%)	64 (69%)		
39	19 (27%)	7 (32%)	26 (28%)		
40	3 (4%)	0 (0%)	3 (3%)		
Sex				0.002	0.96
Female	33 (46%)	11 (50%)	44 (47%)		
Male	38 (54%)	11 (50%)	49 (53%)		
Birth weight (g)				2.58	0.46
2000-2500	13 (18%)	8 (36%)	21 (23%)		
2500-3000	26 (37%)	12 (55%)	38 (41%)		
3000-3500	16 (23%)	10 (45%)	26 (28%)		
3500-4000	8 (11%)	1 (5%)	9 (10%)		
Breastfeeding within one hour				35.14	<0.05
Yes	70 (99%)	10 (45%)	80 (86%)		
No	1 (1%)	12 (55%)	13 (14%)		
Mode of delivery				5.05	0.08
LSCS	56 (79%)	13 (59%)	69 (74%)		
FTNVD	14 (20%)	7 (32%)	21 (23%)		
Assisted NVD	1 (1%)	2 (9%)	3 (3%)		

To assess the practical significance and strength of association between each variable and NICU admission, effect sizes were calculated using Cramér’s V. The results are summarized in Table 3. Most variables demonstrated negligible or small effect sizes. Specifically, gestational age (χ² = 1.08, V = 0.108), birth weight (χ² = 2.58, V = 0.166), and mode of delivery (χ² = 5.05, V = 0.233) showed small to small-moderate effects. Sex (χ² = 0.002, V = 0.005) and meconium-stained liquor (χ² = 0.00, V = 0.000) showed virtually no association. In contrast, early initiation of breastfeeding (within 1 hour) exhibited a large and clinically meaningful effect size (χ² = 35.14, V = 0.615), indicating a strong association with the likelihood of NICU admission (Table 3).

**Table 3:** Effect size calculations (Cramér’s V) for study variables This table presents effect size calculations using Cramér’s V for the association between various clinical variables and NICU admission. The original chi-squared (χ²) test statistics, sample size (n), and degrees of freedom (df) are included. The final column provides an interpretation of the effect size magnitude based on standard guidelines (e.g., small, moderate, and large). NICU, neonatal intensive care unit

Variable	χ²	n	df (k-1)	Cramér’s V	Interpretation
Gestational age	1.08	93	1	0.108	Small effect
Sex	0.002	93	1	0.005	Negligible
Meconium	0	93	1	0	None
Birth weight	2.58	93	1	0.166	Small effect
Breastfeeding <1 hour	35.14	93	1	0.615	Large effect
Mode of delivery	5.05	93	1	0.233	Small-moderate

## Discussion

Newborns undergo rapid physiological changes at birth, and failure to establish effective air breathing can lead to respiratory distress, affecting up to 7% of term infants. This distress contributes to significant morbidity in approximately 15% of term infants admitted to NICUs [7]. An increasing number of term infants are being admitted to the NICU due to respiratory distress [8]. Therefore, there is a pressing need to explore interventions that can prevent NICU admission in this population.

CPAP, a noninvasive respiratory support method, is now widely used to prevent and treat respiratory distress, aiming to reduce reliance on invasive mechanical ventilation. CPAP provides positive airway pressure to spontaneously breathing infants throughout the respiratory cycle. In preterm infants born before 33 weeks’ gestation, early CPAP reduces the need for mechanical ventilation and lowers the risk of mortality or chronic lung disease [9]. By maintaining positive airway pressure, CPAP improves FRC, respiratory effort, and gas exchange, thereby enhancing oxygenation and ventilation [10]. During the transition after birth, lung aeration facilitates clearance of lung fluid, establishes FRC, and increases pulmonary blood flow. The effect of positive pressure on lung volume depends on lung elasticity, which increases with gestational age, and chest wall elasticity, which decreases with gestational age [11]. Thus, CPAP is particularly beneficial for preterm infants with RDS.

Term neonates delivered via cesarean section often have higher pulmonary liquid-to-air ratios, increasing susceptibility to respiratory distress. Delayed fluid clearance contributes to the pathophysiology of this condition. PEEP has been shown to prevent airway liquid re-entry during expiration in animal models [12]. CPAP may reduce secondary lung injury by preventing atelectrauma, surfactant deficiency, oxygen-related injury, and pulmonary hypertension. Additionally, CPAP prevents airway collapse, decreases intrapulmonary shunting, and promotes alveolar recruitment [13].

Some recent studies, however, have reported an increased risk of pneumothorax in late preterm and term infants receiving DR-CPAP in the delivery room [5]. While CPAP helps establish FRC in fluid-filled newborn lungs, its benefits are more pronounced in preterm infants with surfactant deficiency. Physiological differences between term and preterm infants, such as higher surfactant levels and lung compliance in term infants, raise concerns regarding the indiscriminate application of higher CPAP pressures, creating a dilemma about its use in term neonates.

In our study, DR-CPAP produced favorable outcomes: 71 of 93 neonates (76%) recovered within 30 minutes, and only 22 required NICU admission. Among those admitted, just seven required additional CPAP support. This indicates that most neonates responded well to initial respiratory support in the delivery room, with only a small proportion needing further intervention in the NICU. No complications were associated with DR-CPAP, suggesting its safety and efficacy when applied judiciously. Most NICU stays were less than 24 hours, reflecting short-term requirements for advanced respiratory support. Importantly, no neonates required mechanical ventilation, emphasizing the effectiveness of DR-CPAP and other noninvasive support methods in managing respiratory distress in term neonates.

In this single-center experience, DR-CPAP stabilized 71 (76%) term neonates with mild-to-moderate respiratory distress within the first 30 minutes of life, thereby reducing NICU admissions. Among neonates requiring NICU care, only a small proportion needed further CPAP, and none required mechanical ventilation, highlighting the potential of early noninvasive support.

These findings align with broader literature on CPAP in preterm infants, where early CPAP reduces mechanical ventilation needs and improves survival. However, prior studies in term or near-term infants have raised concerns. For example, Sandall et al. (2025) reported that reductions in DR-CPAP use were correlated with decreased pneumothorax in infants ≥36 weeks [14]. Similarly, Smithhart et al. (2019) found that DR-CPAP was associated with higher odds of pneumothorax in infants 35-42 weeks (OR 5.5) [5].

In our cohort, the absence of pneumothorax or air-leak complications may reflect careful protocol standardization (PEEP 5 cm H₂O, FiO₂ 30%), oversight by NRP-trained providers, and prompt monitoring. This suggests that when used appropriately, DR-CPAP can be safe in term neonates.

Limitations

This study has several limitations. The primary limitation is its retrospective, single-center design, which relied on existing medical records and lacked a concurrent control group (no non-DR-CPAP arm), limiting causal inference and generalizability. Additionally, while the sample size provided adequate power for primary outcomes, it was insufficient to detect rare adverse events such as pneumothorax, limiting conclusions about safety. Finally, the study focused on short-term outcomes, preventing assessment of long-term respiratory or neurodevelopmental effects. Future research should adopt prospective designs with comparator arms and include robust regression analyses and CIs to strengthen conclusions and evaluate long-term impacts.

## Conclusions

DR-CPAP was associated with stabilization of term neonates with mild-to-moderate respiratory distress in our single-center cohort. Our findings suggest a potential reduction in NICU admissions without observed adverse events in this population. These results provide preliminary support for the feasibility and effectiveness of early noninvasive ventilation in the delivery room in similar clinical settings. Larger prospective studies are needed to confirm its safety, efficacy, and long-term outcomes, as this study does not establish causation.
